# The role of CXCL16 in atherosclerosis: from mechanisms to therapy

**DOI:** 10.3389/fimmu.2025.1555438

**Published:** 2025-05-26

**Authors:** Yue Liu, Xintao Tian, Chunyan Jia, Xinrui Cheng, Changxing Cui, Cuiping Li, Shaonan Yang

**Affiliations:** ^1^ Department of Neurology, The Affiliated Hospital of Qingdao University, Qingdao, Shandong, China; ^2^ Department of Urology, The Affiliated Hospital of Qingdao University, Qingdao, Shandong, China; ^3^ Department of Emergency Internal Medicine, The Affiliated Hospital of Qingdao University, Qingdao, Shandong, China; ^4^ Department of Critical Care Medicine, The Affiliated Hospital of Qingdao University, Qingdao, Shandong, China

**Keywords:** CXCL16, chemokine, CXCR6, atherosclerosis, mechanism

## Abstract

Atherosclerosis (AS), as the primary pathological basis of cardiovascular and cerebrovascular diseases, is closely associated with chemokines in its occurrence and progression. CXCL16 establishes a new link between chemokines and AS. We briefly introduced the structural characteristics of CXCL16 and its specific receptor CXCR6, as well as related signaling pathways. Furthermore, the significant role of CXCL16 in the progression of AS was elaborated from the perspective of pathological mechanisms and signal pathways. Meanwhile, we objectively evaluated the potential arterial protective effects of CXCL16. Finally, we discussed various novel therapeutic strategies to alleviate AS by targeting the inhibition of CXCL16 and its regulatory pathways. This review systematically summarizes the multifaceted roles of CXCL16 in AS, providing theoretical foundations and research insights for the precise prevention and treatment of AS.

## 1 Introduction

Cardiovascular and cerebrovascular diseases, as global health issues, are characterized by high mortality, high disability, and high recurrence rates (1). Atherosclerosis (AS) is the main underlying cause of these diseases (2). Major risk factors for AS include hypertension (3), diabetes (4), hyperlipidemia (5), smoking (6), and excessive alcohol consumption (7). These factors synergistically contribute to the occurrence and progression of AS (8). In recent years, numerous studies have demonstrated that chemokines play an important role in the pathological process of AS, influencing all stages of its development (such as endothelial cell injury, inflammatory cell recruitment, and smooth muscle cell proliferation) (9).

Chemokines are small secreted proteins whose core function is to mediate the directional migration of immune cells through chemotaxis (10). According to the arrangement pattern of conserved cysteine residues at the N-terminus, chemokines can be classified into four subfamilies: CXC, CC, CX3C and C (11). Among these, the CXC subfamily is the most diverse. According to whether or not they contain the ELR (glutamate-leucine-arginine) protein sequence, CXC chemokines can be further divided into functional subgroups with either pro-angiogenic (ELR+) or anti-angiogenic (ELR-) activity (12) (**Table 1**). CXCL16 is a member of the CXC family (13). CXCL16 is generally considered an independent risk factor for AS (14, 15). However, a small number of studies suggest that CXCL16 may have protective effects against AS (16, 17).

**Table 1:** Chemokines in the CXC family and their role in atherosclerosis.

Classification	Chemokines	Receptors	The role in atherosclerosis	Mechanism	References
ELR+	CXCL1	CXCR2	promotion	Proliferation, migration and Tube formation of endothelial cells	(18)
	CXCL2	CXCR2	promotion	Mediates inflammatory response	(19)
	CXCL3	CXCR2	promotion	Inflammation and oxidative stress	(20)
	CXCL5	CXCR1/CXCR2	inhibition/ promotion	–	(21, 22)
	CXCL6	CXCR1/CXCR2	promotion	–	(23)
	CXCL7	CXCR2	promotion	Acts as a chemotactic factor for neutrophils	(24)
	CXCL8	CXCR2/CXCR3	promotion	Mediates inflammatory response	(25)
ELR-	CXCL4	CXCR3	promotion	Mediates inflammatory response and inhibit the function of scavenger receptors	(26)
	CXCL9	CXCR3	promotion	Recruits activated T cells	(27)
	CXCL10	CXCR3	promotion	Recruits activated T cells	(27)
	CXCL11	CXCR3	promotion	Recruits activated T cells	(27)
	CXCL12	CXCR4	promotion	Dyslipidemia, inflammation, neointimal hyperplasia, angiogenesis, and insulin resistance	(28)
	CXCL13	CXCR5	promotion	Reduces the secretion of protective IgM	(29)
	CXCL14	–	promotion	Smooth muscle cell migration and proliferation	(30)
	CXCL16	CXCR6	promotion/ inhibition	See below	

This article elucidates the mechanisms of CXCL16 in AS and its associated signaling, while also summarizing relevant targeted therapies.

## 2 CXCL16 and its receptor

CXCL16 was first cloned in 2000 by Shimaoka et al. (31). Its structure primarily consists of four functional domains: a transmembrane region, a short cytoplasmic domain, an extracellular N-terminal chemokine domain, and a glycosylated mucin-like stalk (32). Along with CX3CL1, it is currently recognized as one of only two known transmembrane chemokines (33). Beyond its classical chemokine functions, CXCL16 exhibits two additional biological roles as an adhesion molecule and a scavenger receptor (34). CXCL16 exists in two different forms: membrane binding (mCXCL16) and soluble CXCL16 (sCXCL16) (35, 36). The generation of these forms depends on proteolytic cleavage of the extracellular domain by the metalloproteinase ADAM10 (37, 38). These two molecular forms demonstrate distinct biological functions (35, 36).

**Figure 1:**
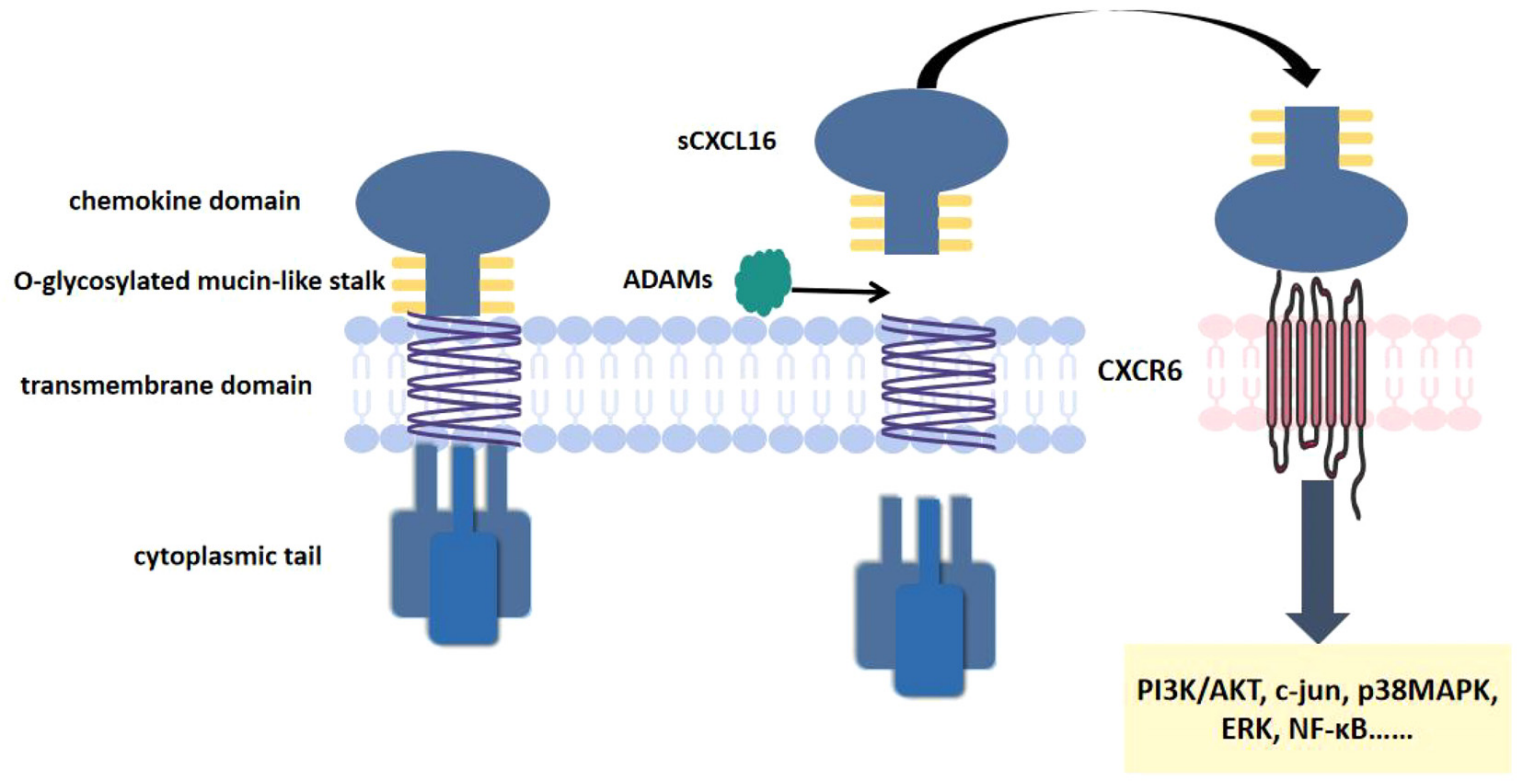
CXCL16 and CXCR6 structure. CXCL16 consists of a transmembrane region, a short cytoplasmic domain, an extracellular N-terminal chemokine domain. CXCR6 is a seven-transmembrane GPCR. CXCL16 binds to CXCR6, activating downstream signaling pathways.

mCXCL16 functions as both an adhesion molecule and a scavenger receptor (39). It primarily mediates the recognition and uptake of oxidized low-density lipoproteins (oxLDL) and phosphatidylserine (PS) (40). Therefore, it was initially named the scavenger receptor for phosphatidylserine and oxidized lipoproteins (SR-PSOX) (41). CXCR6 is a seven-transmembrane G protein-coupled receptor (GPCR) (42). It was originally identified as a co-receptor for human immunodeficiency virus (HIV) in CD4+ and CD8+ T cells (43, 44). It is also known as CD186, Bonzo, STRL, or TYMSTR (42, 45). CXCR6 is the specific receptor for sCXCL16. Through their specific binding, sCXCL16 induces proliferation and migration of CXCR6-expressing cells, while activating downstream signaling pathways (including NF-κB, PI3K/AKT, MAPK pathways) to regulate the occurrence and progression of AS (32, 46). (**Figure 1**).

## 2 The pro-atherosclerotic effect of CXCL16

**Figure 2:**
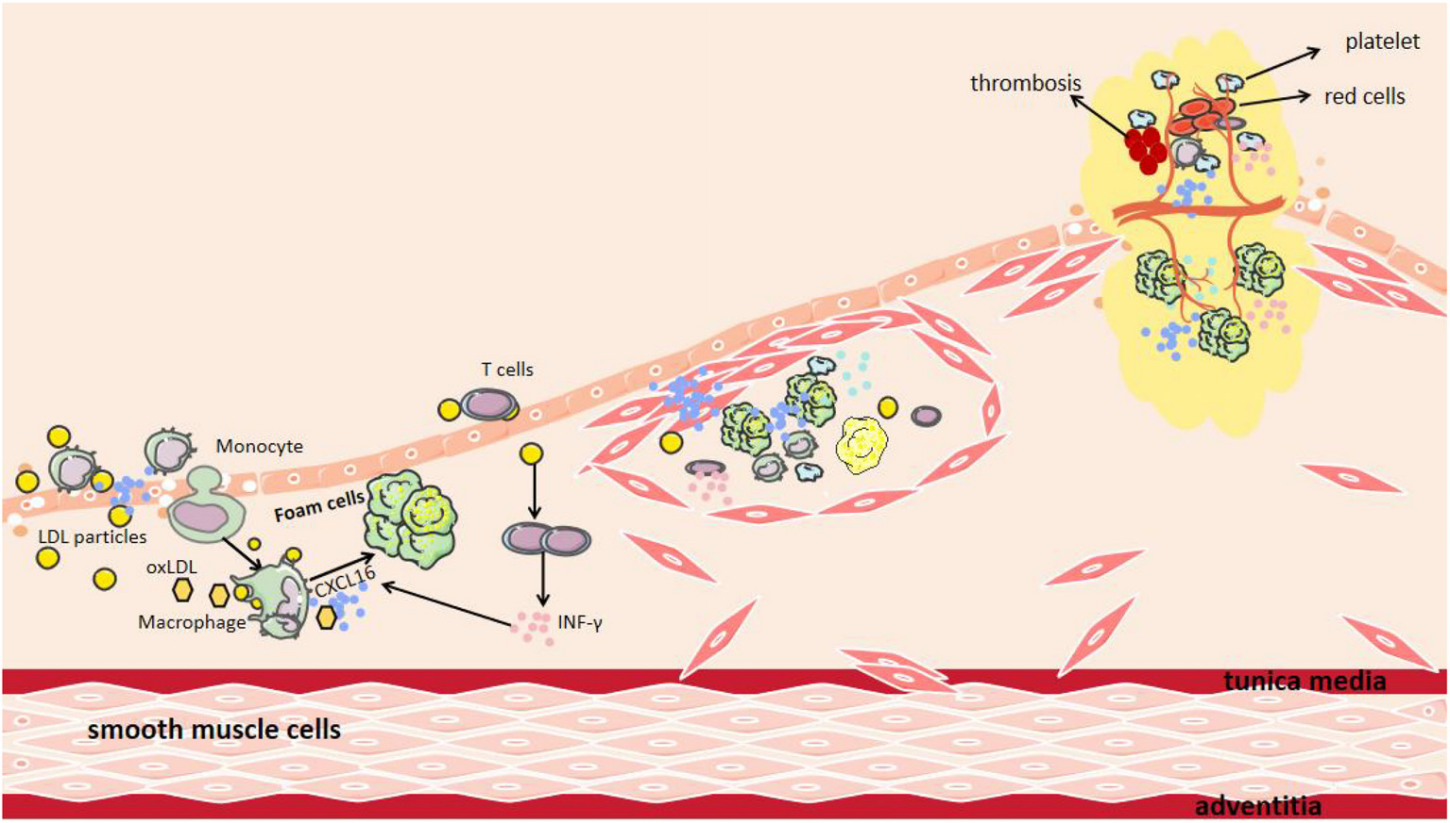
The Role of CXCL16 in AS. After endothelial cell injury, CXCL16 on the endothelial cells surface mediates the recruitment and adhesion of both inflammatory cells and pro-inflammatory cytokines to the damaged surface. Recruited macrophages subsequently infiltrate the intimal layer, where CXCL16 facilitates their phagocytosis of LDL and oxidized LDL (oxLDL). Additionally, CXCL16 acts as a scavenger receptor, providing receptors for oxLDL. INF-γ produced by T cells increases the production of CXCL16. Furthermore, CXCL16 can promote the aggregation of other blood cells and the formation of new blood vessels, leading to plaque instability.

**Figure 3:**
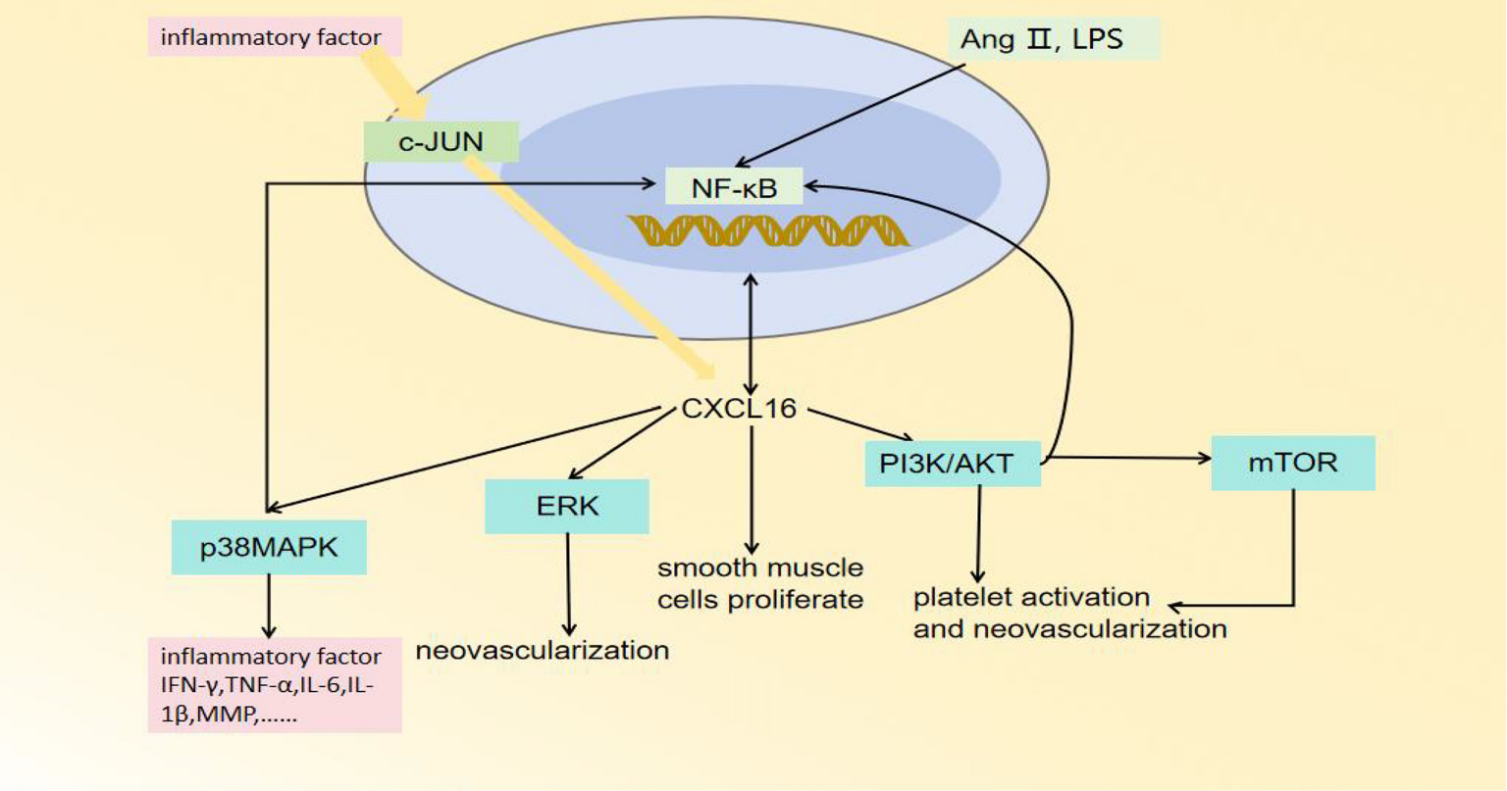
The regulatory pathways of CXCL16.

**Figure 4:**
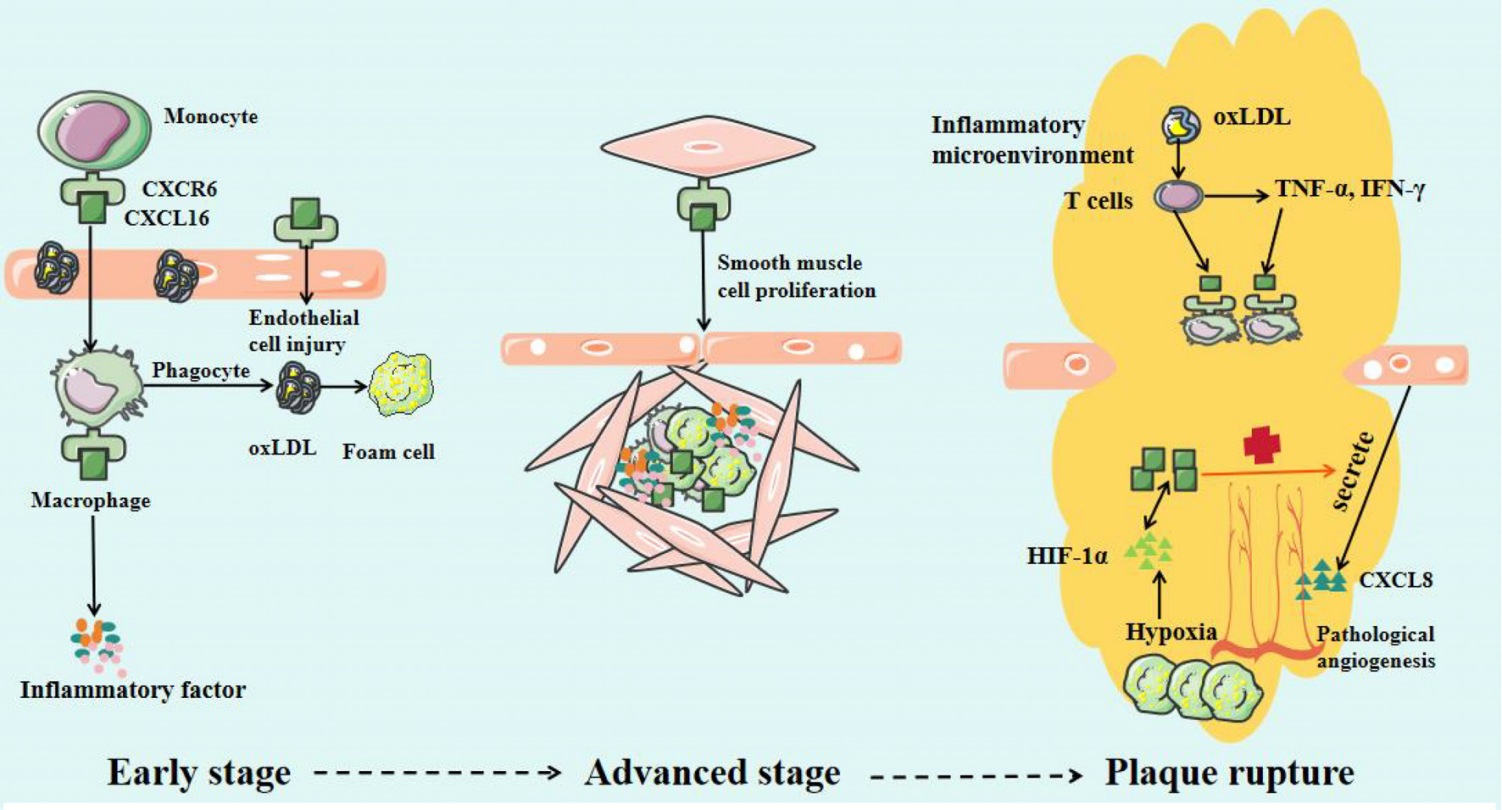
CXCL16/CXCR6 Axis in Different Cells and at Different Stages.

CXCL16 plays an important role in the occurrence, progression, and plaque destabilization of AS. During the early disease stage, CXCL16 promotes inflammatory cell infiltration and foam cell formation, accelerating lipid deposition and vascular endothelial injury (47). In the advanced stage, it further stimulates smooth muscle cell proliferation and migration, inducing intimal thickening and fibrous cap formation (48). At the late stage, sustained inflammatory responses, blood cell aggregation, and pathological neovascularization can lead to fibrous cap thinning, increasing plaque rupture risk (**Figure 2**) (49). Mechanistic studies reveal that CXCL16 mediates vascular inflammatory responses, intimal remodeling, and angiogenesis through regulation of PI3K/AKT, MAPK, and NF-κB signaling pathways, thereby multi-dimensionally promoting AS progression (**Figure 3**) (50). Thus, a comprehensive understanding of CXCL16’s pathological role (**Figure 4**) and molecular mechanisms in AS will provide crucial theoretical foundations for developing targeted intervention strategies for AS.

### 2.1 CXCL16 induces vascular inflammatory response

Chemokines drive sustained low-level chronic inflammatory responses by recruiting inflammatory cells and factors to the lesion sites (51). This persistent inflammatory state not only facilitates atherosclerotic plaque formation but also leads to plaque destabilization and rupture (52) (**Table 2**).

**Table 2:** The Mechanism of CXCL16 Chemotaxis of Various Cells, Cytokine Secretion, and Involvement in Atherosclerosis.

Cell type	Cytokines	Effect on atherosclerosis	References
Monocyte-Macrophage	MMPs	Plaque Instability	(73, 75)
	CCL4 CCL5	Chemotaxis	(54)
	IL-6	Induces Inflammation	(68)
	IL-1β	Induces Inflammation	(68)
	TNF-α	Induces Inflammation	(68)
T cells	INF-γ	Activates Macrophages	(63)
	TNF-α	Amplifies Inflammatory Response	(65)
	IL-17A	Induces Inflammation	(69)
	PD-1	Induces Inflammation	(76–78)
Endothelial Cells	HIF-α	Induces Angiogenesis	(79)
	VEGF	Induces Angiogenesis	(80, 81)
	CXCL8	Induces Angiogenesis	(80, 81)
	ICAM-1	Adhesion	(82)
	MMPs	Plaque Instability	(83)
Smooth Muscle Cells	IFN-γ	Uptakes of oxLDL	(48)
	MMPs	Smooth Muscle Cell Proliferation	(84)
	ICAM-1	Induces Inflammation	(85)

CXCL16 specifically chemoattracts various CXCR6-expressing immune cells, including monocytes/macrophages, T cells, NK cells, invariant natural killer T cells and plasma cells (46). In the early stages of AS, when the vascular endothelial barrier is destroyed, CXCL16 recruits monocytes from circulation to the subendothelial space through chemotaxis, facilitating their differentiation into macrophages (53). In the murine model of myocardial infarction, CXCL16-mediated activation of NF-κB and p38MAPK pathways drives upregulation of CCL4 and CCL5, resulting in amplified monocyte recruitment and subsequent aggravation of cardiac injury (54). In human umbilical vein endothelial cells (HUVECs) and macrophages, LPS and the proinflammatory cytokine TWEAK can induce CXCL16 production through NF-κB signaling pathways (55–57). It is noteworthy that CXCL16 serves not only as a chemokine but also plays an equally important role as a scavenger receptor (58). It can recognize and mediate the phagocytosis of oxLDL by macrophages, promoting the formation of foam cells, which is a critical step in the development of AS (59, 60).

With the continuous development of AS, the continuous accumulation of oxLDL can activate T cells, promoting their secretion of pro-inflammatory factors such as IFN-γ and TNF-α (61, 62). Notably, these cytokines (particularly TNF-α and IFN-γ) can further stimulate T cells to produce CXCL16, continuously recruiting macrophages, and maintaining inflammatory microenvironment (63–65). Once T cells infiltrate the plaques, they may undergo clonal proliferation and secrete large amounts of inflammatory cytokines (66). This process ultimately leads to plaque destabilization and even rupture (67). CXCL16 can promote the secretion of inflammatory factors (including IL-6, IL-1β, VCAM-1, ICAM-1 and IL-17A) in macrophages and T cells of plaques through activation of the p38 MAPK signaling pathway, thereby exacerbating plaque instability and contributing to adverse cardiovascular events (68–72). Furthermore, excessive activation of the CXCL16/CXCR6 axis upregulates the expression of matrix metalloproteinases (MMPs), leading to the abnormal degradation of elastin and collagen in the vascular extracellular matrix and promoting calcium salt deposition. These pathological changes ultimately result in fibrous cap thinning and rupture (68, 73). This finding has been experimentally validated in ApoE-/- mice: lentivirus-mediated CXCL16 overexpression significantly upregulated inflammatory mediators (including MMPs, CCL2, VCAM-1, and TNF-α) and markedly aggravated plaque instability (74).

### 2.2 CXCL16 induces intimal thickening

In the advanced stages of AS, vascular intimal hyperplasia not only exacerbates the retention of lipoprotein in the intima but also accelerates significantly the progression of AS (86). Vascular smooth muscle cells (VSMCs) play a crucial role in phenotypic switching and functional dysregulation (87). Current studies have confirmed that CXCL16 promotes VSMCs proliferation and migration by multiple mechanisms (48, 60, 88). Bysani Chandrasekar et al. demonstrated that CXCL16 enhanced aortic smooth muscle cell proliferation and migration in a PI3K/AKT-dependent manner (88). Similarly, Chandrasekar, B. et al. found that CXCL16 increased intercellular adhesion and stimulated VSMC proliferation by activating the NF-κB signaling pathway (60). Additionally, the uptake of oxLDL induced by IFN-γ depends on the upregulation of CXCL16 expression in VSMCs (48). Inflammatory cytokines boost CXCL16 expression by inducing c-Jun binding to the CXCL16 promoter, thereby promoting VSMC proliferation and contributing to AS (88, 89). In addition, aging VSMCs exhibit increased expression of chemokines (such as CXCL16), adhesion molecules (such as ICAM-1), and innate immune receptors (such as Toll-like receptors 4) (82, 85). These changes collectively establish a persistent pro-inflammatory microenvironment that further promotes the progression of AS. Although the precise mechanisms of CXCL16 in VSMCs remain unclear, targeting this chemokine may offer a new therapeutic strategy to mitigate post-angioplasty restenosis (88).

### 2.3 CXCL16 induces angiogenesis

As the lesion progresses, pathological thickening of the vascular wall leads to luminal stenosis, significantly reducing local tissue perfusion and inducing a hypoxic state (90). The abnormal accumulation of lipids and the formation of necrotic core in plaques create a vicious cycle, continuously stimulating the upregulation of hypoxia-inducible factor-1α (HIF-1α) expression (91). This chronic hypoxic microenvironment activates pro-angiogenic signaling pathways, inducing the formation of pathological neovascularization within plaques. These structurally fragile neovessels not only increase the risk of intraplaque hemorrhage but also significantly elevate the potential for plaque rupture (92).

In 2009, Zhuge, X. et al. revealed that CXCL16 is a angiogenic factor with multifunctional regulatory properties (93). CXCL16 promotes pathological neovascularization in a dose-dependent manner (79). Immunohistochemical analysis found that CXCL16 was strongly expressed in endothelial cells of pathological neovascularization (94). CXCL16 promotes angiogenesis through multiple mechanisms. Firstly, the hypoxic microenvironment of atherosclerotic plaques induces HIF-1α, which in turn upregulates CXCL16 expression (95). Conversely, CXCL16 secreted by HUVECs further enhances HIF-1α-mediated vascular endothelial growth factor (VEGF) production by activating the PI3K/AKT signaling pathway, forming a pro-angiogenic vicious cycle (96, 97). Additionally, prior studies confirmed that CXCL16 markedly enhanced the proliferative capacity, chemotactic motility, and vascular network formation of HUVECs *in vitro* by activating the ERK pathway (93). Secondly, CXCL16 can induce endothelial cells to produce the potent pro-angiogenic factor CXCL8/IL-8 (98), indirectly promoting angiogenesis through this paracrine mechanism (80, 81). Thirdly, CXCL16 also induces the expression of MMPs in endothelial cells. These enzymes degrade the extracellular matrix and release stored pro-angiogenic factors, ultimately contributing to plaque instability (99).

## 3 The protective effect of CXCL16 against AS

A few studies have reported that CXCL16 has a protective effect against AS. Aslanian, A. M. et al. found that atherosclerotic plaque burden was unexpectedly increased in LDLR-/- mouse models with CXCL16 gene knockout. The authors attributed this phenomenon primarily to impaired scavenger receptor function caused by CXCL16 deficiency, which subsequently reduced the efficiency of apoptotic cell clearance. Additionally, this study demonstrated that CXCL16 knockout significantly reduced oxLDL binding and internalization by macrophages *in vitro (* 16). These findings markedly contradict previous research conclusions regarding the pro-atherogenic roles of classical scavenger receptors such as SR-A and CD36 (100, 101). We speculate that the bidirectional regulatory effects of CXCL16 stem from its dual functional properties (chemokine *vs* scavenger receptor). In different pathological microenvironments, when one function becomes dominant, corresponding phenotypic characteristics emerge (16). In addition, the choice of animal models and the impact of gene knockout may affect further investigations. Similarly, a study reported that the level of CXCL16 were reduced in patients of coronary AS (17). However, this study only included a small number of patients with stable or unstable angina. And the results of this study were overturned in a larger cohort study (102). Van Lieshout A. W.,et al. questioned the results of this study (103). Based on current research, we believe that the conclusions of this study require further exploration.

## 4 Targeted therapy

Atherosclerotic cardiovascular disease currently stands as the leading global cause of mortality (104). Although lipid-lowering medications can effectively reduce blood lipid and inflammatory cytokine levels, some individuals still exhibit residual cardiovascular risk, highlighting the need for further therapeutic interventions (105). As key regulators of leukocyte migration, chemokines play a central role in immune surveillance and inflammatory responses. In recent years, drug development targeting the chemokine system has become a major focus in the treatment of inflammatory diseases (106). Throughout the entire process of AS development, the chemokine network participates in regulating multiple pathological pathways, making it a highly promising therapeutic target (9). Among these, CXCL16 and its signaling pathway play significant roles by mediating inflammatory cell infiltration, regulating the proliferation of vascular endothelial and smooth muscle cells, and influencing angiogenesis (47–49). Although early studies suggested that CXCL16 may possess dual regulatory properties, recent evidence consistently indicated that its pro-atherogenic effect dominated. Considering the limitations of previous studies proposing a “protective role” hypothesis, we propose that selective inhibition of CXCL16 and its signaling pathway may serve as an effective strategy to delay the progression of AS (**Table 3**).

**Table 3:** The anti-atherosclerotic effects of various drugs and compounds at the chemokine molecular level.

Drugs or molecules	Mechanisms	References
Anti-CXCL16 neutralizing antibodies	Inhibition of monocyte infiltration and inflammatory cytokine secretion	(54, 109, 110)
P2X7R inhibitor	Reduction of CXCL6 expression and lipid accumulation	(119, 120)
PCSK9 inhibition monoclonal antibody	Limits leukocyte-endothelial cell interactions	(121)
Pertussis toxin	Inhibits cell adhesion and smooth muscle proliferation	(60)
Compound 17	Reduction of CXCR6 expression.	(125)
Compound 81	Reduction of CXCR6 expression.	(125)
Resveratrol	Reduce ADAM10-mediated cleavage of CXCL16 and T cell recruitment	(126)
Statins	Reduces the release of ADAM10	(116)
Aspirin	Reduces endotoxin production in macrophages and oxidative stress in the body	(55)
Ticagrelor	Reduces platelet activation, adhesion, and aggregation	(37)
Irbesartan	Reduces the production of inflammatory factors	(118)
Docosahexaenoic acid (DHA)	Reduces the production of inflammatory factors	(132)
MAPKK inhibitor	Production of CXCL16 and the tube formation process of endothelial cells	(93)

### 4.1 Anti-atherosclerotic effects by inhibiting CXCL16, CXCR6 and proteases

Compared with non-atherosclerotic tissues, atherosclerotic plaques contain significantly higher levels of inflammatory cells, inflammatory factors, and lipid deposits (107, 108). Studies showed that targeting CXCL16 could effectively modulate this pathological process. For instance, in murine models of myocardial infarction, administration of anti-CXCL16 neutralizing antibodies inhibits monocyte infiltration and improve cardiac function after myocardial infarction (54). Similarly, in murine models of glomerulonephritis, anti-CXCL16 neutralizing antibodies reduced the expression of IL-4 and IL-10 (109). In a murine model of Salmonella enterica serovar Enteritidis infection, anti-CXCL16 neutralizing antibodies reduced the expression of IFN-γ (110). Additionally, lentivirus-mediated CXCL16 knockdown can inhibit macrophage transformation into foam cells and reduce lipid deposition in the arterial walls of ApoE-/- mice (111, 112). These findings collectively indicated that targeting CXCL16 could reduce the accumulation of inflammatory cells, cytokines, and lipids, thereby mitigating the development of AS.

In addition to the direct inhibition of CXCL16, studies have found that the promoter region of CXCL16 contains the binding site of FOXO3, and targeting FOXO3 could reduce the expression of CXCL16 (113). In addition, the basic amino acid residues in the CXCL16 chemokine domain are critical for its function. Point mutation of basic amino acid plays a key role in CXCL16 function. Disrupting these key residues can attenuate the pro-atherogenic effects of CXCL16 (114, 115). The presence of these molecular recognition elements demonstrates the druggability of this target. Currently, some available drugs (including aspirin, ticagrelor, irbesartan, PCSK9-blocking monoclonal antibody, P2X7R inhibitor A438079) can reduce the release of CXCL16 and inflammatory responses (37, 55, 116–121).

Targeting the specific receptor CXCR6 of CXCL16 can also attenuate the development of AS. Studies found that CXCR6 knockout in mice inhibited monocyte infiltration into vascular walls, reduced inflammatory response and myocardial ischemia-reperfusion injury (122). Elena Galkina et al. revealed that CXCR6-deficient ApoE-/- mice reduced T cell and macrophage infiltration in aortic walls along with suppressed production of pro-inflammatory cytokine IFN-γ (123). The Gi receptor inhibitors (pertussis toxin) exert protective effects by both blocking cell adhesion and inhibiting aortic smooth muscle proliferation (60). These findings collectively indicated that targeting CXCR6 produced effects comparable to those observed with CXCL16 inhibition. Postea, O. et al. found that homocysteine could enhance CXCR6-mediated lymphocyte recruitment, thereby promoting the progression of AS (124). This suggests that homocysteine-lowering medications potentially reduce inflammatory cell accumulation. Some compounds (such as compound 81 and compound 17) have been found to reduce the expression of CXCR6 (125). With their favorable oral bioavailability, these compounds represent promising candidates for next-generation anti-atherosclerotic therapies.

ADAM10 serves as the key protease mediating the conversion of mCXCL16 to sCXCL16 (126). Gough, P. J. et al. showed that knocking down ADAM10 reduced this constitutive shedding of CXCL16. ADAM10 inhibitors, such as resveratrol, can effectively block the proteolytic processing of CXCL16 (116, 126). A small molecule compound (GI254023X) has been identified as ADAM10 inhibitor that reduces the release of sCXCL16 (127). Simvastatin, a commonly used clinical drug, has also been found to inhibit the activity of ADAM10 (116).

### 4.2 Anti-atherosclerotic effects by inhibiting the regulatory mechanisms of CXCL16

CXCL16 exerts anti-inflammatory effects by modulating multiple signaling pathways including NF-κB, PI3K/AKT, and MAPK, thereby attenuating AS progression (50). Various pharmacological agents, such as NF-κB inhibitors (JSH-23, SN50), aspirin, rapamycin, PI3K/AKT inhibitors, and irbesartan, have been demonstrated to downregulate CXCL16 expression (55, 118, 128, 129). Various NF-κB inhibitors have proven effective in reducing inflammatory cell infiltration and suppressing atherosclerotic plaque formation (54, 55). Aspirin can inhibit the nuclear translocation of the NF-κB p65 subunit, thus reducing the progression of AS (130). The PI3K inhibitor (LY294002 or wortmannin), AKT inhibitor (SH-6) and JNK inhibitor (SP600125) can reduce platelet adhesion and smooth muscle cell proliferation (60, 79, 131). The p38 inhibitors and ERK inhibitors (PD98059) inhibit HUVEC proliferation, migration, tube formation and HIF-1α expression. Notably, the HIF-1α selective inhibitor (PX-12) not only inhibits these biological processes but also suppresses CXCL16 production (79).

In this section, we have highlighted that numerous existing drugs can effectively reduce CXCL16 or modulate its receptor, protease, and related signaling pathways. This drug repurposing strategy offers the advantage of accelerating clinical validation while significantly reducing development costs. However, it should be noted that currently there are no drugs specifically targeting CXCL16. Therefore, the development of CXCL16-specific probes and advanced nanodelivery technologies has become an imperative research direction.

## 5 Conclusion

CXCL16 is an important chemokine and immune regulator, which is widely expressed in various cells such as endothelial cells, monocytes, macrophages, and T cells. Previous studies showed that CXCL16 played a complex dual regulatory role in the development of AS. In this review, we summarize the key pathological mechanisms and related signaling pathways of CXCL16 in promoting AS, while also providing an objective evaluation of its potential protective effects. Based on current evidence, we propose that the pro-inflammatory and pro-atherogenic effects of CXCL16 dominate in AS. Therefore, targeted inhibition of CXCL16 represents a promising therapeutic approach for AS. Although the development of CXCL16-targeted drugs still faces numerous challenges such as target selectivity and optimization of drug delivery methods, advances in both mechanistic understanding and novel drug delivery technologies are expected to lead to breakthrough progress. These studies may not only yield more effective treatments but also uncover new intervention targets, thereby opening new avenues for the prevention and treatment of AS.
